# Imported Monkeypox from International Traveler, Maryland, USA, 2021 (Response)

**DOI:** 10.3201/eid2808.220830

**Published:** 2022-08

**Authors:** Varea Costello, Madeleine Sowash, Aahana Gaur, Michael Cardis, Helena Pasieka, Glenn Wortmann, Sheena Ramdeen

**Affiliations:** Walter Reed National Military Medical Center, Bethesda, Maryland, USA (V. Costello);; Medstar Washington Hospital Center, Washington, D.C., USA (M. Sowash, A. Gaur, M. Cardis, H. Pasieka, G. Wortmann, S. Ramdeen)

**Keywords:** Monkeypox, zoonoses, travel medicine, global health, Orthopoxvirus, viruses, United States

**In Response**: We appreciate Minhaj et al. for their correspondence regarding our experience treating an imported case of monkeypox from an international traveler (*1*). Their letter reaffirms the instructive points of our case: monkeypox poses a diagnostic challenge because its clinical presentation shares features with a variety of additional infectious diseases, including varicella zoster virus (the infection we initially suspected), and prompt coordination with public health officials is critical for diagnosing, treating, and mitigating secondary spread.

Since our report of monkeypox in November 2021, there have been outbreaks of monkeypox throughout multiple countries (*2*), including one case identified in the United States (*3*). Monkeypox had never previously been diagnosed in several of these countries and, remarkably, in only 1 of these cases (*4*) was there a history of travel to a monkeypox-endemic country, in direct contrast to nearly all prior cases that have been reported outside of Africa (*5*–*7*), which were epidemiologically linked to a monkeypox-endemic region.

In our case report (*8*), we had concluded that monkeypox had become clinically relevant within the confines of a travel-related illness. However, the additional cases diagnosed since November 2021 strongly suggest that community transmission is now occurring, and a history of travel to a monkeypox-endemic country is no longer prerequisite to contracting this disease. Community prevalence rates remain unknown, so healthcare providers should consider monkeypox in any patient who manifests with fever and lymphadenopathy accompanied by a disseminated vesicular, pustular, or umbilicated rash. Under those conditions, the provider should immediately initiate infection control and contact public health authorities. Monkeypox is an emerging zoonotic disease with incompletely appreciated clinical features, and healthcare providers should be made aware of its increasingly widespread incidence. 
